# Supplementary material for: Evaluating the impact of injury prevention interventions in child and adolescent sports using the RE-AIM framework and CERT: A systematic review
Source: PLoS One. 2023 Jul 21;18(7):e0289065. doi: 10.1371/journal.pone.0289065 (PMC10361493; doi:10.1371/journal.pone.0289065)
Supplement: S1 Table — (DOCX) [file pone.0289065.s003.docx]

| **Table S1** - Online search strategy – also available on PROSPERO (Registration number CRD42021272847) | | | | | | | |
| --- | --- | --- | --- | --- | --- | --- | --- |
| Search engine | Search terms |  |  |  |  |  |  |
| EBSCOhost – SportDiscus PsychINFO and Medline | "injury prevention" OR "prevention of injur*" OR "prevention program OR "Neuromuscular"  OR "prevention programme" OR "neuromuscular training"  OR "balance training" OR "conditioning program"  OR "exercise program" OR "conditioning programme"  OR "proprioception training"  OR "proprioceptive training" | AND | injur*  OR "sports injur*"  OR "athletic injur*"  OR "injury risk" | AND | sport* OR athlet*  OR handball  OR baseball OR basketball OR football OR soccer  OR golf OR gymnastics  OR hockey OR running  OR swimming OR Netball  OR volleyball OR cycling  OR squash OR Tennis  OR Trampolining OR Rugby OR Cricket OR Dance  OR polo OR wrestling OR baseball OR softball OR golf | AND | "young" OR  "adolescent"  OR "adolesc*"  OR "youth"  OR "juvenile"  OR "teen*"  OR "school aged"  OR "high school"  OR "secondary  school" |
| The Cochrane Library | 1   #1  MeSH descriptor: [Athletic Injuries] this term only MeSH descriptor: [Athletic Injuries] this term only   MeSH 719 | | | | | | |
|  | 2   #2  MeSH descriptor: [Child] explode all trees MeSH descriptor: [Child] explode all trees   MeSH 57420 | | | | | | |
|  | 3   #3  MeSH descriptor: [Youth Sports] explode all trees MeSH descriptor: [Youth Sports] explode all trees   MeSH 13 | | | | | | |
|  | 4   #4  MeSH descriptor: [Sports] explode all trees MeSH descriptor: [Sports] explode all trees   MeSH 16041 | | | | | | |
|  | 5   #5  (adult*):ti,ab,kw (adult*):ti,ab,kw  in Trials  S Limits 691395 | | | | | | |
|  | 6   #6  ("injury reduction" OR "injury prevention" OR "neuromuscular training" OR "prevention program" OR "prevention programme") | | | | | | |
|  | 7   #7  (injur* OR "sports injur*" OR "athletic injur*" OR "injury risk") | | | | | | |
|  | 8   #8  (sport* OR athlet* OR handball OR baseball OR basketball OR football OR soccer OR golf OR gymnastics OR hockey OR running OR swimming OR Netball OR volleyball  OR cycling OR squash OR Tennis OR Trampolining OR Rugby OR Cricket OR Dance OR polo OR wrestling OR baseball OR softball OR golf) | | | | | | |
|  | 9   #9  (child* OR adolescen* OR youth* OR juvenile OR kid*) (child* OR adolescen* OR youth* OR juvenile OR kid*) | | | | | | |
|  | 10    #10  (#6) AND (#7 OR #1) AND (#8 OR #3 OR #4) AND (#9 OR #2) NOT (#5) (#6) AND (#7 OR #1) AND (#8 OR #3 OR #4) AND (#9 OR #2) NOT (#5)   in Trials  Limits 180 | | | | | | |
| SCOPUS | ( TITLE-ABS-KEY ( ( *"injury reduction"*  OR  *"injury prevention"*  OR  *"neuromuscular training"*  OR  *"prevention program"*  OR  *"prevention programme"* ) )  AND  ALL ( ( *injur**  OR  *"sports injur*"*  OR  *"athletic injur*"*  OR  *"injury risk"* ) )  AND  ALL ( ( *sport**  OR  *athlet**  OR  *handball*  OR  *baseball*  OR  *basketball*  OR  *football*  OR  *soccer*  OR  *golf*  OR  *gymnastics*  OR  *hockey*  OR  *running*  OR  *swimming*  OR  *netball*  OR  *volleyball*  OR  *cycling*  OR  *squash*  OR  *tennis*  OR  *trampolining*  OR  *rugby*  OR  *cricket*  OR  *dance*  OR  *polo*  OR  *wrestling*  OR  *baseball*  OR  *softball*  OR  *golf* ) )  AND  ALL ( ( *child**  OR  *adolescent*  OR  *youth**  OR  *juvenile*  OR  *kid**  OR  *adolescen** ) )  AND NOT  TITLE-ABS-KEY ( *adult** ) )  AND  ( LIMIT-TO ( DOCTYPE ,  *"ar"* ) )  AND  ( LIMIT-TO ( LANGUAGE ,  *"English"* ) )  AND  ( LIMIT-TO ( SRCTYPE ,  *"j"* ) ) | | | | | | |
| Pubmed | ((("injury prevention"[Title/Abstract] OR "prevention of injur*"[All Fields] OR "prevention program"[Title/Abstract] OR  "Neuromuscular"[Title/Abstract] OR "prevention programme"[Title/Abstract] OR "neuromuscular training"[Title/Abstract]  OR "balance training"[Title/Abstract] OR "conditioning program"[Title/Abstract] OR "exercise program"[Title/Abstract]  OR "conditioning programme"[Title/Abstract] OR "proprioception training"[Title/Abstract] OR "proprioceptive training"[Title/Abstract]) AND ("injur*"[Title/Abstract] OR "sports injur*"[All Fields] OR "athletic injur*"[All Fields] OR "injury risk"[Title/Abstract]  OR "soft tissue injuries"[MeSH Terms] OR "sprains and strains"[MeSH Terms] OR "tendons/pathology"[MeSH Terms]  OR "tendon injuries"[MeSH Terms] OR "fractures, bone"[MeSH Terms] OR "fractures, cartilage"[MeSH Terms] OR "musculoskeletal system/injuries"[MeSH Terms] OR "athletic injuries"[MeSH Terms]) AND ("sport*"[Title/Abstract] OR "athlet*"[All Fields]  OR "exercise"[Title/Abstract] OR "physical activity"[Title/Abstract] OR "game"[Title/Abstract] OR "recreation"[Title/Abstract]  OR "training"[Title/Abstract] OR "handball"[All Fields] OR "baseball"[MeSH Terms] OR "basketball"[MeSH Terms]  OR "football"[MeSH Terms] OR "soccer"[MeSH Terms] OR "golf"[MeSH Terms] OR "gymnastics"[MeSH Terms]  OR "hockey"[MeSH Terms] OR "racquet sports"[MeSH Terms] OR "running"[MeSH Terms] OR "swimming"[MeSH Terms]  OR "volleyball"[MeSH Terms] OR "exercise"[MeSH Terms] OR "Netball"[All Fields] OR "cycling"[All Fields] OR "squash"[All Fields]  OR "Tennis"[All Fields] OR "track and field"[All Fields] OR "Trampolining"[All Fields] OR "Rugby"[All Fields] OR "Cricket"[All Fields]  OR "Dance"[All Fields] OR "diving"[All Fields] OR "polo"[All Fields] OR "Fencing"[All Fields] OR "Ice Skating"[All Fields]  OR "Martial Arts"[All Fields] OR "wrestling"[All Fields] OR "climbing"[All Fields] OR "sailing"[All Fields] OR "skiing"[All Fields]  OR "triathlon"[All Fields]) AND ("child*"[Title/Abstract] OR "young"[Title/Abstract] OR "adolescent"[MeSH Terms]  OR "adolesc*"[Title/Abstract] OR "youth"[Title/Abstract] OR "juvenile"[Title/Abstract] OR "teen*"[Title/Abstract]  OR "school aged"[Title/Abstract] OR "high school"[Title/Abstract] OR "secondary school"[Title/Abstract]))  NOT ("Adult"[Title/Abstract] OR "systematic review"[Title] OR "meta-analysis"[Title/Abstract])) AND ((fha[Filter])  AND (english[Filter])) | | | | | | |
