# Supplementary material for: Evaluating the impact of injury prevention interventions in child and adolescent sports using the RE-AIM framework and CERT: A systematic review
Source: PLoS One. 2023 Jul 21;18(7):e0289065. doi: 10.1371/journal.pone.0289065 (PMC10361493; doi:10.1371/journal.pone.0289065)
Supplement: S2 Table — (DOCX) [file pone.0289065.s004.docx]

| **Table S2** - Consensus on Exercise Reporting Template Example - Adapted from Slade et al., (2016) | | |
| --- | --- | --- |
|  |  |  |
| Author/year |  |  |
| Title |  |  |
| Reviewer and date |  |  |
|  |  |  |
|  |  | SCORE |
| Item | Description | Yes =1, No = 0, N/A |
|  |  |  |
| 1 | Exercise equipment reported in detail |  |
| 2 | Qualifications/expertise/training reported in detail |  |
| 3 | Individual or group exercises described |  |
| 4 | Supervised or unsupervised described |  |
| 5 | Adherence measured/reported and how |  |
| 6 | Motivation strategies described |  |
| 7a | Rules for progression described |  |
| 7b | Exercise progression described |  |
| 8 | Description of **each exercise** in detail |  |
| 9 | Description of home program content N/A if none |  |
| 10 | Describe non-exercise components N/A if none |  |
| 11 | Describe if any adverse events occurred |  |
| 12 | What setting were they performed described |  |
| 13 | DETAILED description of the intervention |  |
| 14a | Describe exercises are generic or tailored to the individual |  |
| 14b | Describe how they were tailored (N/A if generic) |  |
| 15 | Describe decision for starting level |  |
| 16a | Describe how fidelity was assessed |  |
| 16b | Describe whether it was delivered as planned |  |
|  |  |  |
|  |  | Total score = |
