# Supplementary material for: Evaluating the impact of injury prevention interventions in child and adolescent sports using the RE-AIM framework and CERT: A systematic review
Source: PLoS One. 2023 Jul 21;18(7):e0289065. doi: 10.1371/journal.pone.0289065 (PMC10361493; doi:10.1371/journal.pone.0289065)
Supplement: S3 Table — (DOCX) [file pone.0289065.s005.docx]

| **Table S3 – All reviewed studies and additional papers (Original trial in bold).** Details of the reviewed studies, RE-AIM scores for each domain, total RE-AIM and CERT scores | | | | | | | | | | | | |  |  |
| --- | --- | --- | --- | --- | --- | --- | --- | --- | --- | --- | --- | --- | --- | --- |
|  |  | Study details | | |  |  | RE-AIM elements | | | | | |  | Consensus on Exercise Reporting Template (CERT) score (%) |
|  |  |  |  |  |  |  | Score given as - number of criteria satisfied/total applicable criteria | | | | | |  |  |
| **Original trial** and additional papers | Study Design | Sample size (n), Age (years), Sex | Setting, country | Sport | Intervention type, Length NMT = Neuromuscular training |  | Reach | Effectiveness | Adoption - Setting and Delivery agent | Implementation | Maintenance - Individual and Setting | Total RE-AIM score (%) |  |  |
| **Achenbach et al., 2018**[83] | Block RCT | 279, 13-18, Male and Female | Sports club, Germany | Handball | NMT - 15 min |  | 1/4 | 2/5 | 1/8 | 1/5 | 0/9 | 16 |  | 41 |
| **Åkerlund et al., 2020**[95] - Åkerlund et al., 2021[96] - Åkerlund et al., 2023[97] | Cluster RCT | 471, 12-17, Male and Female | Sports club, Sweden | Floorball | NMT (*Knee Control*) - 20 min |  | 2/4 | 4/5 | 4/8 | 5/5 | 2/9 | 55 |  | 75 |
| **Al Attar et al., 2022**[98] | Cluster RCT | 780, 7-13, Male | Sports club, Saudi Arabia | Soccer | FIFA 11+ Kids, 20 min |  | 3/4 | 3/5 | 2/8 | 2/5 | 0/9 | 32 |  | 59 |
| **Asgari et al., 2022**[99] | RCT | 90, 12-18, Male | Sports Club, Iran | Soccer | NMT (FIFA 11+/Modified F11), 20 min |  | 1/4 | 3/5 | 2/8 | 4/5 | 0/9 | 32 |  | 31 |
| **Asker et al., 2022**[100] | Cluster RCT | 627, 14-19, Male and Female | School, Sweden | Handball | NMT (Knäkontroll/Knee Control/Shoulder control) - 15 min |  | 3/4 | 4/5 | 2/8 | 4/5 | 0/9 | 42 |  | 71 |
| **Azuma et al., 2020**[49] | RCT | 124, 16.2 (SD ± 0.8), Male | School, Japan | Soccer | Static stretching - 20-30 min |  | 2/4 | 3/5 | 0/4 | 1/5 | 2/9 | 35 |  | 19 |
| **Barboza et al., 2019**[101] | Quasi-Experimental | 291, 10-17, Male and Female | Sports club, Netherlands | Field hockey | NMT (*WUP*) - 12 min |  | 2/4 | 2/5 | 3/8 | 2/5 | 0/9 | 29 |  | 59 |
| **Barden et al., 2022a**[57] - Barden et al., 2021a[91] -Barden et al., 2021b[28] -Barden et al., 2022b[60] | Quasi-Experimental | 659,U12-U19, Male, | Schools, UK | Rugby | NMT (*Activate*) - 20 min |  | 3/4 | 5/5 | 5/8 | 4/5 | 7/9 | 77 |  | 38 |
| **Collard et al., 2010a**[50] - Collard et al., 2010b[102] | Cluster RCT | 2210, 10-12, Male and Female | Primary school, Netherlands | Various/PA | Education + NMT - 5 min |  | 4/4 | 4/5 | 4/8 | 2/5 | 3/9 | 55 |  | 41 |
| **Emery et al., 2005**[71] | Cluster RCT | 127, 15.6-16.1, Male and Female | School/home, Canada | Various | Balance - 20 min |  | 2/4 | 3/5 | 1/4 | 2/5 | 0/9 | 30 |  | 65 |
| **Emery et al., 2007**[103] | Cluster RCT | 920, 12-18, Male and Female | School/home, Canada | Basketball | Balance(home) - 20 min, Balance (practice) - 5 min |  | 2/4 | 3/5 | 2/8 | 3/5 | 0/9 | 32 |  | 53 |
| **Emery and Meeuwisse, 2010**[104] - Marshall et al., 2016[105] | Cluster RCT | 744, 13-18, Male and Female | Sports club, Canada | Soccer | NMT and home balance - 15 min (NMT), 20 min (balance) |  | 2/4 | 3/5 | 3/8 | 1/5 | 1/9 | 32 |  | 61 |
| **Emery et al., 2020**[74] - Richmond et al., 2020[78] - Räisänen et al., 2022[81] | Cluster RCT | 1067, 11-16, Male and Female | School, Canada | Various | NMT (*iSprint*) - 15 min |  | 2/4 | 4/5 | 4/8 | 5/5 | 1/9 | 52 |  | 71 |
| **Emery et al., 2022**[13] | Quasi-Experimental | 307, 11-18, Male and Female | School and Sports club, Canada | Basketball | NMT (*SHRed*) - 20 min |  | 2/4 | 3/5 | 4/8 | 3/5 | 1/9 | 42 |  | 71 |
| **Foss et al., 2018**[106] | Cluster RCT | 474, 14 (SD ± 1.7), Female | School, US | Soccer, volleyball, basketball | NMT- (*CORE*) - 15-25 min |  | 0/4 | 2/5 | 0/8 | 1/5 | 0/9 | 10 |  | 38 |
| **Hasebe et al., 2020**[72] | Cluster RCT | 259, 15-18, Male | School, Japan | Soccer | Nordic hamstring exercise - time not reported |  | 2/4 | 3/5 | 2/8 | 2/5 | 0/9 | 29 |  | 69 |
| **Heidt et al., 2000**[107] | RCT | 300, 14-18, Female | School, US | Soccer | Running/Plyometrics - 20 min |  | 0/4 | 1/5 | 0/8 | 1/5 | 0/9 | 7 |  | 13 |
| **Hewett et al., 1999**[51] | Prospective controlled cohort study | 1263, 14-18, Male and Female | School, US | Soccer, volleyball, basketball | NMT - 60-90 min |  | 0/4 | 3/5 | 0/8 | 1/5 | 2/9 | 19 |  | 50 |
| **Hilska et al., 2021a**[59] - Hilska et al., 2021b[20] | Cluster RCT | 1403, 9-14, Male and Female | Sports club, Finland | Soccer | NMT - 20 min |  | 4/4 | 4/5 | 2/8 | 4/5 | 3/9 | 55 |  | 75 |
| **Hislop et al., 2017**[12] - Hislop et al., 2016[108] | Cluster RCT | 3188, 14-18, Male | School, UK | Rugby | NMT (*Activate*) - 20 min |  | 2/4 | 4/5 | 3/8 | 2/5 | 0/9 | 35 |  | 73 |
| **Kiani et al., 2010**[16] | Quasi-Experimental | 1506, 12.7-18.6, Female | Sports club, Sweden | Soccer | NMT (*HarmoKnee*) - 20-25 min |  | 1/4 | 2/5 | 2/8 | 3/5 | 3/9 | 36 |  | 53 |
| **LaBella et al., 2011**[56] | Cluster RCT | 1492, 16.2 (SD ± 1.06), Female | School, US | Soccer, basketball | NMT - 20 min |  | 3/4 | 3/5 | 3/4 | 5/5 | 0/9 | 52 |  | 63 |
| **Malliou et al., 2004**[109] | Quasi-Experimental | 100, 16-17, Male | Sports club, Greece | Soccer | Balance - 20 min |  | 0/4 | 2/5 | 0/8 | 1/5 | 0/9 | 10 |  | 0 |
| **Mandelbaum et al., 2005**[17] | Prospective controlled cohort study | 2757, 14-18, Female | Sports club, US | Soccer | NMT (*PEP program*) - 15 min |  | 1/4 | 1/5 | 1/8 | 0/5 | 1/9 | 13 |  | 38 |
| **McGuine and Keene, 2006**[110] | RCT | 765, 15-17, Male and Female | School, US | Soccer, basketball | Balance 10 min |  | 1/4 | 2/5 | 1/8 | 1/5 | 0/9 | 16 |  | 35 |
| **Mendez-Rebolledo et al., 2021**[52] | RCT | 22, 11-18, Male and Female | Sports club, Chile | Running | NMT - 30 min |  | 1/4 | 3/5 | 0/4 | 2/5 | 0/9 | 22 |  | 25 |
| **Olsen et al., 2005**[111] | Cluster RCT | 1837, 16-17, Male and Female | Sports club, Norway | Handball | NMT 15-20 min |  | 2/4 | 1/5 | 2/8 | 4/5 | 0/9 | 29 |  | 56 |
| **Pfeiffer et al., 2006**[112] | Prospective controlled cohort study | 2016, 15-18, Male and Female | School, US | American football, basketball, Soccer, lacrosse | NMT (*KLIP)* - time not reported |  | 1/4 | 2/5 | 0/8 | 1/5 | 0/9 | 13 |  | 50 |
| **Rahlf and Zech, 2020**[113] - Rahlf et al., 2020[114] | Cluster RCT | 342, 15.5 (SD ± 1.6), Male | Sports club, Germany | Soccer | NMT (*FIFA 11+*) - 10/20 min |  | 0/4 | 5/5 | 1/8 | 3/5 | 0/9 | 29 |  | 44 |
| **Richmond et al., 2015**[115] | Cluster RCT | 725, 11-15, Male and Female | School, Canada | Various | NMT - 15 min |  | 2/4 | 5/5 | 2/8 | 3/5 | 0/9 | 37 |  | 75 |
| **Rössler et al., 2018**[15] - Beaudouin et al., 2018[116] | Cluster RCT | 3895, 10.7 (SD ± 1.4), Male and Female | Sports club, Germany, Switzerland, Czech Republic, Netherlands | Soccer | NMT - 15-20 min (*FIFA 11+)* |  | 3/4 | 4/5 | 3/8 | 3/5 | 0/9 | 42 |  | 63 |
| **Sakata et al., 2018**[117] | Prospective controlled cohort study | 305, 8-11, Male and Female | Sports club, Japan | Baseball | Stretching/muscle strength - 20 min |  | 1/4 | 3/5 | 1/8 | 2/5 | 4/9 | 36 |  | 53 |
| **Sakata et al., 2019**[73] | RCT | 237, 9-11, Male and Female | Sports club, Japan | Baseball | Stretching/muscle strength - 20 min |  | 0/4 | 4/5 | 1/8 | 2/5 | 0/9 | 23 |  | 59 |
| **Scase et al., 2006**[48] | Quasi-Experimental | 723, 17, Male | Sports club, Australia | Aussie football | Landing practice - 30 min |  | 0/4 | 2/5 | 0/8 | 1/5 | 0/9 | 10 |  | 31 |
| **Slauterbeck et al., 2019**[45] – Krug et al., 2021[80] | Cluster RCT | 3611, 14-18, Male and Female | School, US | American football, basketball, soccer, lacrosse | NMT - 20 min |  | 2/4 | 3/5 | 3/8 | 5/5 | 0/9 | 42 |  | 81 |
| **Soligard et al., 2009**[82] - Soligard et al., 2010[18] | Cluster RCT | 1892, 13-17, Female | Sports club, Norway | Soccer | NMT (*FIFA 11*) - 20 min |  | 3/4 | 5/5 | 4/8 | 4/5 | 0/9 | 52 |  | 69 |
| **Steffen et al., 2008**[118] | Block RCT | 2092, 15.4 (SD ± 0.8), Female | Sports club, Norway | Soccer | NMT (*FIFA 11*) - 20 min |  | 3/4 | 4/5 | 1/8 | 3/5 | 0/9 | 36 |  | 63 |
| **Steffen et al., 2013a**[119] - Steffen et al., 2013b[19] | Cluster RCT | 385, 13-18, Female | Sports club, Canada | Soccer | NMT (*FIFA 11+*) - 20 min |  | 1/4 | 4/5 | 2/8 | 5/5 | 0/9 | 39 |  | 69 |
| **Veith et al., 2021**[120] | RCT | 65, 12-16, Male | Sports club, Austrailia | Soccer | NMT - time not reported |  | 0/4 | 4/5 | 0/4 | 3/5 | 0/9 | 26 |  | 41 |
| **Verhagen et al., 2023**[14] | Cluster RCT | 518, 10-17, Male and Female | Sports club, The Netherlands | Volleyball | NMT (VolleyVeilig) - 15 min |  | 2/4 | 2/5 | 3/4 | 3/5 | 0/9 | 37 |  | 25 |
| **Walden et al., 2012**[58] - Hägglund et al., 2009[121] -Hägglund et al., 2013[89] - Lindblom et al., 2014[85] - Åman et al., 2018[90] | Cluster RCT | 4564, 14-18, Female | Sports club, Sweden | Soccer | NMT (*Knäkontroll/Knee Control*) - 15 min |  | 2/4 | 4/5 | 4/8 | 3/5 | 5/9 | 58 |  | 69 |
| **Wedderkopp et al., 1999**[122] | Prospective controlled cohort study | 237, 16-18, Female | Sports club, Denmark | Handball | NMT - 10-15 min |  | 0/4 | 2/5 | 0/8 | 0/5 | 0/9 | 7 |  | 6 |
| **Wedderkopp et al., 2003**[123] | Cluster RCT | 163, 14-16, Female | Sports club, Denmark | Handball | Balance 10-15 min |  | 0/4 | 1/5 | 2/8 | 0/5 | 0/9 | 10 |  | 6 |
| **Zarei et al., 2019**[124] | Cluster RCT | 962, 7-14, Male | Sport club, Iran | Soccer | NMT (*FIFA 11+*) - 20 min |  | 1/4 | 3/5 | 4/8 | 2/5 | 0/9 | 32 |  | 69 |
| **Zouita et al., 2016**[125] | RCT | 52, 13-14, Male | Sports club, Tunisia | Soccer | Muscle strength - 90 min |  | 0/4 | 2/5 | 0/4 | 1/5 | 0/9 | 11 |  | 47 |
|  |  |  |  |  |  |  |  |  |  |  |  |  |  |  |

Additional references in supplementary material

95. Åkerlund I, Waldén M, Sonesson S, Hägglund M. Forty-five per cent lower acute injury incidence but no effect on overuse injury prevalence in youth floorball players (aged 12-17 years) who used an injury prevention exercise programme: Two-armed parallel-group cluster randomised controlled trial. Br J Sports Med. BMJ Publishing Group; 2020;54:1028–35. . DOI 10.1136/bjsports-2019-101295

96. Åkerlund I, Waldén M, Sonesson S, Lindblom H, Hägglund M. High compliance with the injury prevention exercise programme Knee Control is associated with a greater injury preventive effect in male, but not in female, youth floorball players. Knee Surgery, Sports Traumatology, Arthroscopy. Springer Science and Business Media Deutschland GmbH; 2022;30:1480–90. . DOI 10.1007/s00167-021-06644-2

97. Åkerlund I, Sonesson S, Lindblom H, Waldén M, Hägglund M. Perceptions, facilitators, and barriers regarding use of the injury prevention exercise programme Knee Control among players and coaches in youth floorball: a cross-sectional survey study. BMC Sports Sci Med Rehabil. 2023;15:56. . DOI 10.1186/s13102-023-00660-0

98. Al Attar WSA, Bizzini M, Alzahrani H, Alarifi S, Ghulam H, Alyami M, et al. The FIFA 11+ Kids Injury Prevention Program Reduces Injury Rates Among Male Children Soccer Players: A Clustered Randomized Controlled Trial. Sports Health: A Multidisciplinary Approach. 2023;15:397–409. . DOI 10.1177/19417381221109224

99. Asgari M, Nazari B, Bizzini M, Jaitner T. Effects of the FIFA 11+ program on performance, biomechanical measures, and physiological responses: A systematic review. J Sport Health Sci. 2023;12:226–35. . DOI 10.1016/j.jshs.2022.05.001

100. Asker M, Hägglund M, Waldén M, Källberg H, Skillgate E. The Effect of Shoulder and Knee Exercise Programmes on the Risk of Shoulder and Knee Injuries in Adolescent Elite Handball Players: A Three-Armed Cluster Randomised Controlled Trial. Sports Med Open. 2022;8:91. . DOI 10.1186/s40798-022-00478-z

101. Barboza SD, Nauta J, Emery C, Van Mechelen W, Gouttebarge V, Verhagen E. A warm-up program to reduce injuries in youth field hockey players: A quasi-experiment. J Athl Train. National Athletic Trainers’ Association Inc.; 2019;54:374–83. . DOI 10.4085/1062-6050-79-18

102. Collard DCM, Chinapaw MJM, Verhagen EALM, van Mechelen W. Process evaluation of a school based physical activity related injury prevention programme using the RE-AIM framework. BMC Pediatr. 2010;10. . DOI 10.1186/1471-2431-10-86

103. Emery CA, Rose MS, Mcallister JR, Meeuwisse WH. A Prevention Strategy to Reduce the Incidence of Injury in High School Basketball: A Cluster Randomized Controlled Trial. Clin J Sport Med. 2007.

104. Emery CA, Meeuwisse WH. The effectiveness of a neuromuscular prevention strategy to reduce injuries in youth soccer: A cluster-randomised controlled trial. Br J Sports Med. 2010;44:555–62. . DOI 10.1136/bjsm.2010.074377

105. Marshall DA, Lopatina E, Lacny S, Emery CA. Economic impact study: Neuromuscular training reduces the burden of injuries and costs compared to standard warm-up in youth soccer. Br J Sports Med. BMJ Publishing Group; 2016;50:1388–93. . DOI 10.1136/bjsports-2015-095666

106. Foss KDB, Thomas S, Khoury JC, Myer GD, Hewett TE. A school-based neuromuscular training program and sport-related injury incidence: A prospective randomized controlled clinical trial. J Athl Train. National Athletic Trainers’ Association Inc.; 2018;53:20–8. . DOI 10.4085/1062-6050-173-16

107. Heidt RS, Sweeterman LM, Carlonas RL, Traub JA, Tekulve FX. Avoidance of Soccer Injuries with Preseason Conditioning. Am J Sports Med. 2000.

108. Hislop MD, Stokes KA, Williams S, Mckay CD, England M, Kemp SPT, et al. The efficacy of a movement control exercise programme to reduce injuries in youth rugby: A cluster randomised controlled trial. BMJ Open Sport Exerc Med. BMJ Publishing Group; 2016;2. . DOI 10.1136/bmjsem-2015-000043

109. Malliou P, Gioftsidou A, Pafis G, Beneka A, Godolias G. Proprioceptive training (balance exercises) reduces lower extremity injuries in young soccer players. J Back Musculoskelet Rehabil. IOS Press; 2004.

110. McGuine TA, Keene JS. The effect of a balance training program on the risk of ankle sprains in high school athletes. American Journal of Sports Medicine. 2006;34:1103–11. . DOI 10.1177/0363546505284191

111. Olsen OE, Myklebust G, Engebretsen L, Holme I, Bahr R. Exercises to prevent lower limb injuries in youth sports: Cluster randomised controlled trial. Br Med J. 2005;330:449–52. . DOI 10.1136/bmj.38330.632801.8F

112. Pfeiffer RP, Shea KG, Roberts D, Grandstrand S, Bond L. Lack of Effect of a Knee Ligament Injury Prevention Program on the Incidence of Noncontact Anterior Cruciate Ligament Injury. JOURNAL OF BONE AND JOINT SURGERY. 2006.

113. Rahlf AL, Zech A. Comparison of 10 vs. 20 min neuromuscular training for the prevention of lower extremity injuries in male youth football: A cluster randomised controlled trial. J Sports Sci. Routledge; 2020;38:2177–85. . DOI 10.1080/02640414.2020.1776459

114. Rahlf AL, John C, Hamacher D, Zech A. Effects of a 10 vs. 20-Min Injury Prevention Program on Neuromuscular and Functional Performance in Adolescent Football Players. Front Physiol. Frontiers Media S.A.; 2020;11. . DOI 10.3389/fphys.2020.578866

115. Richmond SA, Kang J, Doyle-Baker PK, Nettel-Aguirre A, Emery CA. A School-Based Injury Prevention Program to Reduce Sport Injury Risk and Improve Healthy Outcomes in Youth: A Pilot Cluster-Randomized Controlled Trial [Internet]. 2015. Available from: www.cjsportmed.com www.cjsportmed.com

116. Beaudouin F, Rössler R, Aus Der Fünten K, Bizzini M, Chomiak J, Verhagen E, et al. Effects of the “11+ Kids” injury prevention programme on severe injuries in children’s football: A secondary analysis of data from a multicentre cluster-randomised controlled trial. Br J Sports Med. BMJ Publishing Group; 2019;53:1418–23. . DOI 10.1136/bjsports-2018-099062

117. Sakata J, Nakamura E, Suzuki T, Suzukawa M, Akaike A, Shimizu K, et al. Efficacy of a Prevention Program for Medial Elbow Injuries in Youth Baseball Players. American Journal of Sports Medicine. SAGE Publications Inc.; 2018;46:460–9. . DOI 10.1177/0363546517738003

118. Steffen K, Myklebust G, Olsen OE, Holme I, Bahr R. Preventing injuries in female youth football - A cluster-randomized controlled trial. Scand J Med Sci Sports. 2008;18:605–14. . DOI 10.1111/j.1600-0838.2007.00703.x

119. Steffen K, Meeuwisse WH, Romiti M, Kang J, McKay C, Bizzini M, et al. Evaluation of how different implementation strategies of an injury prevention programme (FIFA 11+) impact team adherence and injury risk in Canadian female youth football players: A cluster-randomised trial. Br J Sports Med. 2013;47:480–7. . DOI 10.1136/bjsports-2012-091887

120. Veith S, Whalan M, Williams S, Colyer S, Sampson JA. Part 2 of the 11+ as an effective home-based exercise programme in elite academy football (soccer) players: a one-club matched-paired randomised controlled trial. Science and Medicine in Football. Taylor and Francis Ltd.; 2021;5:339–46. . DOI 10.1080/24733938.2021.1874616

121. Hägglund M, Waldén M, Atroshi I. Preventing knee injuries in adolescent female football players - Design of a cluster randomized controlled trial [NCT00894595]. BMC Musculoskelet Disord. 2009;10. . DOI 10.1186/1471-2474-10-75

122. Wedderkopp N, Kaltoft M, Lundgaard B, Rosendahl M, Froberg K. Prevention of injuries in young female players in European team handball. A prospective intervention study. Scand J Med Sci Sports. 1999;9:41–7. . DOI 10.1111/j.1600-0838.1999.tb00205.x

123. Wedderkopp N, Kaltoft M, Holm R, Froberg K. Comparison of two intervention programmes in young female players in European handball - with and without ankle disc. Scand J Med Sci Sports. 2003;13:371–5. . DOI 10.1046/j.1600-0838.2003.00336.x

124. Zarei M, Abbasi H, Namazi P, Asgari M, Rommers N, Rössler R. The 11+ Kids warm-up programme to prevent injuries in young Iranian male high-level football (soccer) players: A cluster-randomised controlled trial. J Sci Med Sport. Elsevier Ltd; 2020;23:469–74. . DOI 10.1016/j.jsams.2019.12.001

125. Zouita S, Zouita ABM, Kebsi W, Dupont G, ben Abderrahman A, ben Salah FZ, et al. Strength Training Reduces Injury Rate in Elite Young Soccer Players During One Season. J Strength Cond Res. 2016;30:1295–307. . DOI 10.1519/JSC.0000000000000920
