# Supplementary material for: Evaluating the impact of injury prevention interventions in child and adolescent sports using the RE-AIM framework and CERT: A systematic review
Source: PLoS One. 2023 Jul 21;18(7):e0289065. doi: 10.1371/journal.pone.0289065 (PMC10361493; doi:10.1371/journal.pone.0289065)
Supplement: S4 Table — (DOCX) [file pone.0289065.s006.docx]

| **Table S4 - Risk of bias assessment of randomised trials using the RoB 2 tool** | | | | |  |  |
| --- | --- | --- | --- | --- | --- | --- |
|  |  | Risk of bias as low/some concerns/high risk | | |  | Overall RoB |
| Author/study | 1. RoB arising from randomisation | 2. RoB due to deviations from intended intervention | 3. RoB due to missing outcome data | 4. RoB in measurement of the outcome | 5. RoB in the selection of reported result | Low/some concerns/high |
| Achenbach et al., 2018[83] | SC | L | L | L | SC | SC |
| Åkerlund et al., 2020[95] | L | L | L | L | L | L |
| Azuma et al., 2020[49] | L | SC | L | SC | SC | SC |
| Al Attar et al., 2022[98] | L | L | L | L | L | L |
| Asgari et al., 2022[99] | L | L | NI/HR | L | SC | HR |
| Asker et al., 2022[100] | L | L | L | L | L | L |
| Collard et al., 2010[50] | L | L | L | SC | SC | SC |
| Emery et al., 2005[71] | L | L | L | L | SC | SC |
| Emery et al., 2007[103] | L | L | L | L | SC | SC |
| Emery and Meeuwisse, 2010[104] | L | SC | L | L | SC | SC |
| Emery et al., 2020[74] | L | L | L | L | L | L |
| Foss et al., 2018[106] | L | L | L | SC | SC | L |
| Hasebe et al., 2020[72] | L | L | L | L | SC | SC |
| Heidt et al., 2000[107] | SC | SC | NI/HR | SC | SC | HR |
| Hilska et al., 2021[59] | L | L | L | L | L | L |
| Hislop et al., 2017[12] | L | L | L | L | L | L |
| LaBella et al., 2011[56] | L | L | L | L | SC | SC |
| McGuine et al., 2006[110] | L | SC | L | SC | SC | SC |
| Mendez-Rebolledo et al., 2021[52] | L | L | L | SC | SC | SC |
| Olsen et al., 2005[111] | L | L | L | L | SC | SC |
| Rahlf and Zech, 2020[113] | L | L | L | L | L | L |
| Richmond et al., 2015[115] | L | L | L | L | SC | SC |
| Rössler et al., 2018[15] | L | L | L | L | L | L |
| Sakata et al., 2019[48] | L | L | L | L | SC | SC |
| Slauterbeck et al., 2019[45] | L | L | L | L | SC | SC |
| Soligard et al., 2009[82] | L | L | L | L | L | L |
| Steffen et al., 2008[118] | L | L | L | L | SC | SC |
| Steffen et al., 2013[119] | L | L | L | L | L | L |
| Veith et al., 2021[120] | L | L | L | L | SC | SC |
| Walden et al., 2012[58] | L | L | L | L | L | L |
| Wedderkopp et al., 2003[123] | SC | SC | NI/HR | SC | SC | HR |
| Zarei et al., 2019[124] | L | L | L | SC | SC | SC |
| Zouita et al., 2016[125] | SC | SC | NH/HR | SC | SC | HR |
