# Supplementary material for: Evaluating the impact of injury prevention interventions in child and adolescent sports using the RE-AIM framework and CERT: A systematic review
Source: PLoS One. 2023 Jul 21;18(7):e0289065. doi: 10.1371/journal.pone.0289065 (PMC10361493; doi:10.1371/journal.pone.0289065)
Supplement: S5 Table — (DOCX) [file pone.0289065.s007.docx]

| **Table S5 - Risk of bias assessment of non-randomised studies using the Robins-I tool** | | | | | | | |  |
| --- | --- | --- | --- | --- | --- | --- | --- | --- |
|  | Risk of bias as low/moderate/serious/critical/no information | | | | | |  |  |
|  | Pre-intervention | | At intervention | | Post-intervention | | | Overall risk of bias |
| Author/study | Bias due to confounding | Bias in selection into the study | Bias in classifications of interventions | Bias due to deviations from intended interventions | Bias due to missing data | Bias in measurement of outcomes | Bias in selection of reported result | low/moderate/serious/critical |
| Barboza et al., 2019[101] | M | L | L | L | L | M | M | Moderate |
| Barden et al., 2022[57] | M | L | L | L | L | M | M | Moderate |
| Emery et al., 2022[13] | L | L | L | L | L | M | M | Moderate |
| Hewett et al., 1999[51] | M | NI | L | NI | L | M | M | Moderate |
| Kiani et al., 2010[16] | M | L | L | L | L | M | M | Moderate |
| Malliou et al., 2004[109] | M | NI | NI | NI | NI/S | M | M | Serious |
| Mandelbaum et al., 2005[17] | M | NI | NI | NI | NI/S | M | M | Serious |
| Pfeiffer et al., 2006[112] | M | NI | L | L | NI/S | M | M | Serious |
| Sakata et al., 2018[117] | M | M | L | NI | NI/S | M | M | Serious |
| Scase et al., 2006[48] | M | L | L | L | L | L | M | Moderate |
| Verhagen et al., 2023[14] | L | L | L | L | L | M | M | Moderate |
| Wedderkopp et al., 1999[122] | L | L | L | NI | NI/S | M | M | Serious |
